# Plant poisonings in livestock in Brazil and South Africa

**DOI:** 10.4102/jsava.v86i1.1200

**Published:** 2015-03-25

**Authors:** Mary-Louise Penrith, Christo J. Botha, Royden C. Tustin

**Affiliations:** 1 Department of Veterinary Tropical Diseases, University of Pretoria, South Africa; 2 TAD Scientific CC, Menlo Park, South Africa; 3 Department of Paraclinical Sciences, University of Pretoria, South Africa

## Abstract

Information on intoxication of livestock by plants in Brazil, in terms of cause, clinical signs and pathology, is compared with information on livestock poisoning by plants in South Africa. Plant poisoning, including mycotoxicosis, is considered to be one of three major causes of death in livestock in Brazil, which is one of the top beef producing countries in the world, with a cattle population of more than 200 million. Cattle production in South Africa is on a more modest scale, but with some 600 species of plants and fungi known to cause toxicity in livestock, as opposed to some 130 species in Brazil, the risk to livestock in South Africa appears to be much greater. The comparisons discussed in this communication are largely restricted to ruminants.

## Communication

The second edition of a book *Plantas Tóxicas do Brasil*, published in 2012 (Tokarnia *et al*. 2012), enables a degree of comparison between plant poisonings and mycotoxicoses in Brazil and southern Africa. The book provides comparisons of some of the plant poisonings described with similar poisonings in Africa, Australia and elsewhere. The aim of the present communication is to make some of the information that is relevant to important plant intoxications in South Africa available in English.

Knowledge of plant poisoning in Brazil has increased rapidly in the last decade. The first edition of the book, published in 2000, described poisoning by 70 plants (Tokarnia, Döbereiner & Peixoto 2000); between 2000 and 2012, the number of plants with proven toxicity to livestock under experimental and natural conditions almost doubled to 130. Brazil has one of the largest cattle populations in the world (211 279 082), as well as significant numbers of sheep (16 789 492) and goats (8 646 463) (FAOSTAT 2012). In 2012, Brazil was ranked the second-largest producer of beef in the world, at 9.3 metric tons per annum (Global Meat News 2014). According to Tokarnia *et al*. (2012), plant poisoning is one of the three most important causes of death in farm animals, the other two being: rabies, transmitted by vampire bats, and epidemic botulism, which is predisposed by phosphate deficiency. Data from the southern states of Brazil indicate that, on average, 10% – 14% of cattle deaths investigated by laboratories involve plant poisoning, and if this is extrapolated to the rest of the country, between 800 000 and 1 120 000 cattle are lost to plant poisoning every year.

Southern Africa has about 600 species of plants known to cause poisoning of livestock, and the total cost of plant poisoning and mycotoxicoses to the livestock industry in South Africa was estimated at ZAR 150 million (Kellerman *et al*. 2005). In contrast to Brazil, South Africa has 13 887 898 cattle, 24 391 112 sheep and 6 141 817 goats (FAOSTAT 2012) and is generally not a major exporter of meat; wool is the most important livestock commodity traded internationally. The expected annual losses due to plant poisoning of domestic ruminants in South Africa in 1996 were 37 665 cattle (10% of expected cattle deaths) and 264 851 small stock per year (Kellerman, Naudé & Fourie 1996).

The reasons for livestock ingesting toxic plants are probably universal: drought, introduction of stock into new areas with different plants, livestock grazing on recently burned pastures, accidental ingestion with other plants, and developing a taste for certain plants despite their toxicity and unpalatability (Botha & Penrith 2008; Tokarnia *et al*. 2012). Differences would, therefore, likely be determined by the availability and abundance of toxic plants, climatic factors and management of both livestock and pastures.

Kellerman *et al*. (1996) identified the six most important plant poisonings of cattle and also of sheep in South Africa; four of these were common to both species and two each were peculiar to one or the other of these species. Three cardiac intoxications, namely: cardiac glycoside poisoning, gousiekte (caused by a polyamine, pavetamine, present in a number of species of the family Rubiaceae) and gifblaar (caused by *Dichapetalum cymosum*, which contains monofluoroacetate) were important in cattle; the first two also affect sheep. Cardiac glycoside-containing plants occur worldwide but significant livestock poisoning has only been reported in southern Africa and, to a much lesser extent, in Australia (Kellerman *et al*. 2005). In Brazil, the only species containing cardiac glycosides listed by Tokarnia *et al*. (2012) is *Nerium oleander*, an ornamental plant of Eurasian origin that is cultivated worldwide. One of the genera of plants incriminated in acute cardiac poisoning in Brazil belongs to the family Rubiaceae, but the toxic principle of one of the species, *Palicourea marcgravii*, has been demonstrated to be monofluoroacetate. This toxin is suspected to be the cause of all acute cardiac plant poisonings in Brazil (Tokarnia *et al*. 2012). Gousiekte in South Africa manifests as acute death, but at necropsy, the heart has characteristic lesions of chronic necrosis and replacement fibrosis, with sudden death provoked by exertion occurring some weeks after initial ingestion of the plant (Prozesky *et al*. 2005). In Brazil, species of *Tetrapterys* (Malpighiaceae) as well as *Ateleia glazoviana* (Fabaceae) may cause sudden death, with chronic heart lesions revealed at necropsy, like gousiekte, but more often the animals develop signs of congestive heart failure before death occurs. Two other syndromes, one involving abortions and the other involving neurological signs including lethargy are associated with poisoning by *A. glazoviana* (Tokarnia *et al*. 2012).

Seneciosis, diplodiosis (caused by the fungus *Stenocarpella maydis*) and poisoning, due to ingestion of *Lantana* spp., occur in both South Africa and Brazil. The intoxications present with the same clinical picture as in South Africa. While seneciosis is well known in Brazil, particularly in the southern states, and appears to show the same pattern as in South Africa, with cattle more often affected than small stock, *Lantana* poisoning appears to be less frequent, and only a single outbreak of diplodiosis has been recorded in Brazil (Tokarnia *et al*. 2012). The other two intoxications of considerable importance to sheep in South Africa are vermeersiekte, caused by *Geigeria* spp., which does not appear to occur in Brazil, and geeldikkop/dikoor caused by *Tribulus terrestris* and *Panicum* spp., respectively (Kellerman *et al*. 2005). The former does not occur in Brazil, and the latter is caused by *Panicum dichotomiflorum* and* Brachiaria decumbens*, a different grass species, in Brazil (Tokarnia *et al*. 2012).

The clinical signs of facial eczema (caused by the fungus *Pithomyces chartarum*) and geeldikkop are due to bile duct occlusion. In facial eczema, inflammatory bile duct occlusion is caused by sporidesmin. In geeldikkop the bile duct obstruction is due to accumulation of birefringent crystals that are produced as a result of steroidal saponins in the plants themselves (Kellerman *et al*. 2005). Although *P. chartarum* is a cosmopolitan saprophyte that has been reported to cause facial eczema in South Africa and some South American countries, following its first description in New Zealand, it has not been diagnosed in Brazil (Tokarnia *et al*. 2012). A poisoning that occurred on *B. decumbens* pastures in Brazil was initially thought to be due to *P. chartarum*; however, investigation revealed that the lesions were caused by birefringent crystals similar to those that typify saponin-induced poisoning by *T. terrestris* and *Panicum* spp., and saponins and sapogenins were subsequently isolated from *B. decumbens* (Tokarnia *et al*. 2012)*. Panicum* intoxication has only been reported in sheep in Brazil (Tokarnia *et al*. 2012), as is the case in South Africa (Kellerman *et al*. 2005).

Other plant poisonings and mycotoxicoses that Brazil and South Africa have in common include those caused by cosmopolitan plants and fungi such as syringa (*Melia azedarach*), castor oil beans (*Ricinus communis*), *Cestrum* spp., *Xanthium* spp., *Amaranthus* spp., *Solanum* spp., *Equisetum* spp., *Pteridium aquilinum* (bracken fern), *Leucaena leucocephala*, *Allium* spp., *Crotalaria* spp., *Vicia villosa, Aspergillus flavus*, *Aspergillus clavatus, Claviceps purpurea, Claviceps paspali* and* Fusarium* spp.

Lysosomal storage diseases that cause neurological disorders and pathology of the central nervous system have been described in both Brazil and southern Africa (Kellerman *et al*. 2005; Tokarnia *et al*. 2012). A disease in South Africa known as ‘maldronksiekte’ is caused by *Solanum tettense* var. *renschii* (formerly *Solanum kwebense*) and is strongly suspected to be a glycoprotein storage disease based on the histopathological lesions (Kellerman *et al*. 2005). In Brazil, several species of *Ipomoea* and *Turbina cordata* (Convolvulaceae) as well as *Sida carpinifolia* (Malvaceae) are responsible for glycoprotein storage disease (Tokarnia *et al*. 2012); one species, *Ipomoea carnea*, has also been reported to cause glycoprotein storage disease in goats in Mozambique (De Balogh *et al*. 1997). In Brazil, *Solanum fastigiatum* var. *fastigiatum* causes neurolipidosis, with clinical signs similar to those produced by *S. tettense* var. *renschii* in South Africa (Tokarnia *et al*. 2012).

Another peculiar neurotoxicity that has been described in both countries is *Prosopis* poisoning. Cattle and goats are poisoned when large quantities of the pods of the highly invasive *Prosopis glandulosa*, also known as mesquite or Suidwesdoring, are ingested. It should be noted that Tokarnia *et al*. (2000, 2012) referred to the plant as *Prosopis juliflora*, a synonym of *P. glandulosa* (Nkonki 2003). Clinically, the animals exhibit excessive rumination, resulting in a greenish staining of the lips, continuous chewing and drooling of saliva. The pathophysiology is ascribed to neuronal vacuolation of the trigeminal nuclei causing denervation atrophy of the masseter and other muscles of mastication (Kellerman *et al*. 2005; Tokarnia *et al*. 2012).

Systemic calcinosis as a result of plant poisoning has not been described in South Africa, but is associated with two plants in Brazil: *Solanum malacoxylon* and *Nierembergia veitchii* (Tokarnia *et al*. 2012).

An interesting poisoning that is reported by Tokarnia *et al*. (2012) is a systemic granulomatous syndrome in dairy cattle caused by ingestion of citrus pulp, which is pelleted as feed for dairy cows in Brazil.

Although plant poisoning of livestock is reported to be important in Brazil, it appears to involve fewer plants than have been reported in South Africa. It is possible that in many parts of Brazil, plant intoxication is under-reported, as many of the poisonings described by Tokarnia *et al*. (2012) occurred in the southernmost provinces, in particular Rio Grande de Sul, where cattle farming is largely commercial and there are good diagnostic facilities; however, the differences between the two countries are likely to be real. The climate of Brazil differs from that of South Africa in that it is warmer and moister, with an average rainfall of 1000 mm – 1500 mm per annum, which is higher than the global average of 860 mm and considerably higher than the average annual rainfall in South Africa, which is 450 mm. Many of the toxic plant species in South Africa are xerophytes that are absent from areas of higher rainfall. Livestock in most of South Africa are exposed to periodic drought conditions that favour the ingestion of toxic plants. Management factors are also important in preventing or precipitating livestock poisoning by plants. In South Africa, a nephrotic syndrome caused by *Nolletia gariepina* was confirmed by dosing trials, but in the field, the intoxication only occurred when cattle that had been penned for periods of 36–48 h without feed were released onto pastures where the plant was abundant (Du Plessis, Prozesky & Botha 2011). It is not known to what extent management plays a role in plant poisoning of livestock in Brazil.
